# Functional, Pharmacogenomic, and Immune Landscapes of Long Non‐Coding RNAs in Cancer

**DOI:** 10.1002/advs.202513414

**Published:** 2025-11-21

**Authors:** Runhao Wang, Mei Luo, Yuan Liu, Jingwen Yang, Yamei Chen, Chengxuan Chen, Lifei Ma, Stephanie Ding, James Wengler, Yong Zang, Bora Lim, Wenbo Li, Liuqing Yang, Chunru Lin, Lixia Diao, Leng Han

**Affiliations:** ^1^ Department of Biostatistics and Health Data Science Indiana University School of Medicine Indianapolis IN 46202 USA; ^2^ Brown Center for Immunotherapy Indiana University School of Medicine Indianapolis IN 46202 USA; ^3^ Center for Computational Biology and Bioinformatics Indiana University School of Medicine Indianapolis IN 46202 USA; ^4^ Department of Psychological and Brain Sciences College of Arts and Sciences Indiana University Bloomington IN 47405 USA; ^5^ Center for Epigenetics and Disease Prevention Institute of Biosciences and Technology Texas A&M University Houston TX 77030 USA; ^6^ Department of Breast Medical Oncology The University of Texas MD Anderson Cancer Center 1515 Holcombe Blvd Houston TX 77030 USA; ^7^ Department of Biochemistry and Molecular Biology The University of Texas Health Science Center Houston Houston TX 77030 USA; ^8^ The Graduate School of Biomedical Sciences The University of Texas MD Anderson Cancer Center Houston TX 77030 USA; ^9^ Department of Molecular and Cellular Oncology The University of Texas MD Anderson Cancer Center Houston TX 77030 USA; ^10^ Department of Bioinformatics and Computational Biology The University of Texas MD Anderson Cancer Center Houston TX 77030 USA

**Keywords:** cancer signaling pathways, drug response, immune checkpoints, immune infiltration, immunotherapy efficacy, irAE, LncRNAs

## Abstract

Long non‐coding RNAs (lncRNAs) are emerging as key regulators in cancer, with significant potential as diagnostic, prognostic, and therapeutic targets. Here, this work systematically analyzes lncRNA associations with targeted therapies and immunotherapies across 33 cancer types using The Cancer Genome Atlas (TCGA) and real‐world datasets. This work identifies 53,173 lncRNA‐pathway associations, millions of lncRNA‐drug response associations (via CancerRxTissue and VAEN), and extensive correlations with immune checkpoints and infiltration. This work further identifies 69 lncRNAs associated with immunotherapy response and 2,611 differentially expressed lncRNAs between low and high objective response rate (ORR) groups. Additionally, two lncRNAs are correlated with immune‐related adverse events (irAEs), and 1,376 lncRNAs exhibited expression differences between cancers with low and high irAE risk. To facilitate further research, this work develops PILNC (https://hanlaboratory.com/PILNC), a comprehensive web portal enabling exploration of lncRNA associations with cancer pathways, drug responses, immune features, and immunotherapy outcomes.

## Graphical abstract

A systematic analysis of 33 cancer types reveals widespread associations between lncRNAs and cancer pathways, drug responses, immune features, and immunotherapy outcomes. The study identifies key lncRNAs linked to therapeutic response and adverse events and introduces PILNC, an interactive web portal for exploring the functional, pharmacogenomic, and immune landscapes of lncRNAs in cancer.

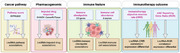

## 1 Introduction

Long non‐coding RNAs (lncRNAs) are RNA molecules longer than 200 nucleotides, characterized by their ability to regulate gene expression and protein function.^[^ ^1^, ^2^ ^]^ They are frequently involved in essential cellular processes such as cell cycle regulation, survival, and immune response.^[^ ^3^, ^4^ ^]^ Despite lacking protein‐coding potential, lncRNAs share many features with cancer genes, acting as either oncogenes (e.g., HOTAIR and NEAT1) or tumor suppressors (e.g., GAS5 and LINC‐PINT) through the modulation of oncogenic molecular networks.^[^ ^5^, ^6^ ^]^ For instance, the tumor‐suppressive lncRNA GAS5, which is significantly downregulated in various cancers, inhibits tumor growth by inducing cell cycle arrest and apoptosis.^[^ ^7^ ^]^ These findings highlight the therapeutic potential of lncRNAs like GAS5, whose roles in cancer regulation provide promising avenues for targeted cancer treatment.

Cancer immunotherapy, a transformative advancement in oncology, harnesses the body's immune system to combat cancer by enhancing or restoring its natural ability to recognize and eliminate malignant cells.^[^ ^8^, ^9^ ^]^ It has become an established clinical modality for treating a wide range of cancers.^[^ ^10^, ^11^, ^12^ ^]^ Immune checkpoint (ICP) targets, such as CTLA‐4, PD‐1, and PD‐L1, have achieved remarkable success across multiple cancers,^[^ ^13^, ^14^, ^15^, ^16^ ^]^ establishing ICP blockade as a cornerstone of modern cancer therapy and an integral component of standard clinical care.^[^ ^17^ ^]^ The tumor immune microenvironment (TIME), shaped by the intricate interplay between the innate and adaptive immune systems, plays a pivotal role in determining immunotherapy efficacy, serving as the dynamic interface where tumors and the immune cells interact.^[^ ^17^ ^]^ Although immunotherapy strengthens the patient's immune system against disease, its activation and disruption of immunologic homeostasis can lead to immune‐related adverse events (irAEs).^[^ ^18^ ^]^ Emerging evidence indicates that lncRNAs, such as LINK‐A, GATA3‐AS1, LIMIT, and NKILA, act as critical regulators of both ICP expression and the TIME, profoundly influence immunotherapy outcomes.^[^ ^19^, ^20^ ^]^ For instance, treatment with LINK‐A antisense oligonucleotides (LNAs) enhanced CD8 T cell infiltration specifically within tumor tissues without altering the distribution of CD8 T cells, macrophages, or MDSCs in normal mammary glands.^[^ ^21^ ^]^ These findings suggest that targeting immune‐related lncRNAs like LINK‐A may enhance antigen presentation and offer promising therapeutic strategies to optimize immunotherapy efficacy. While certain immune factors, such as CD8 T cells, have been identified as predictors of irAE occurrence,^[^ ^22^, ^23^, ^24^ ^]^ the involvement of lncRNAs in irAEs remains largely unexplored. Therefore, a comprehensive understanding of the associations between lncRNAs and the TIME is essential to fully elucidate their role in cancer immunotherapy.

In this study, we leverage multi‐omics data from The Cancer Genome Atlas (TCGA), including transcriptome data and reverse‐phase protein array 500 (RPPA500) data, together with imputed drug response profiles, immune cell abundance estimates, immunotherapy objective response rates (ORRs), and irAE reporting odds ratios (RORs), to investigate the role of lncRNA in modulating the efficacy of cancer therapies. All findings are integrated into a user‐friendly web portal, Pharmacogenomic and Immune Landscape of LncRNA (PILNC, https://hanlaboratory.com/PILNC/), which facilitates the discovery and exploration of lncRNA‐based targets for cancer therapy and immunotherapy.

## 2 Results

### 2.1 Associations between lncRNAs and Cancer Signaling Pathways

**Figure 1:**
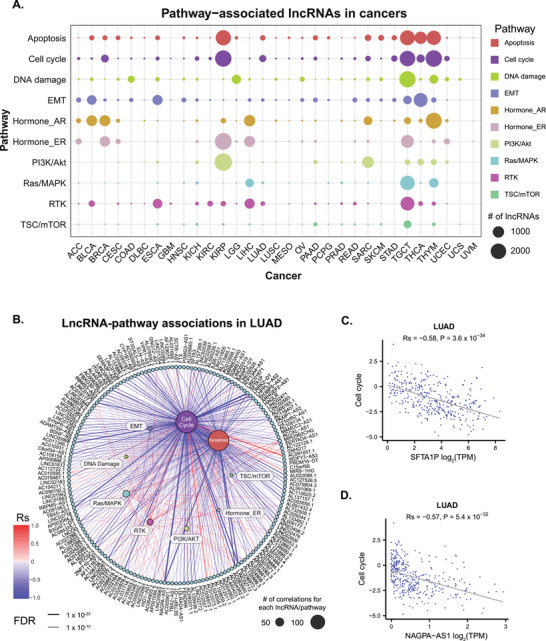
Associations between lncRNAs and cancer signaling pathways. A) The number of lncRNAs associated with ten cancer signaling pathways in various cancer types. B) LncRNA‐pathway associations in lung adenocarcinoma (LUAD). LncRNAs and pathways are represented by dots on the outer and inner circles, respectively. The size of each dot indicates the number of significant correlations associated with the corresponding lncRNA or pathway. Linkage lines between pathways and lncRNAs denote significant correlations, with line colors representing the correlation coefficient (Rs) and line widths corresponding to the FDR value. C) Negative correlation between SFTA1P and the cell cycle pathway score in LUAD. D) Negative correlation between NAGPA‐AS1 and the cell cycle pathway score in LUAD.

To investigate the effects of lncRNAs on cancer signaling pathways, we performed Spearman's correlation analyses between lncRNA expression levels and ten cancer signaling pathway scores derived from RPPA500 data across 33 cancer types from The Cancer Proteome Atlas (TCPA).^[^ ^25^ ^]^ A stringent threshold (|Rs| > 0.3 and FDR < 0.05) was applied to identify significant associations. The number of associations between lncRNAs and pathways varied substantially among cancer types. The top three cancer types with the highest number of lncRNAs associated with pathways were testicular germ cell tumors (TGCT) (n = 11930), kidney renal papillary cell carcinoma (KIRP) (n = 9329), and thymoma (THYM) (n = 7885), whereas lymphoid neoplasm diffuse large B‐cell lymphoma (DLBC) (n = 41), glioblastoma multiforme (GBM) (n = 28), and uveal melanoma (UVM) (n = 5) exhibited the fewest associations (**Figure** **1**A, Table S1, Supporting Information). The proportion of lncRNA associated with various pathways also differed across cancer types. For instance, in breast invasive carcinoma (BRCA), lncRNAs were primarily correlated with cell cycle, apoptosis, hormone ER, and AR pathways, whereas in lung adenocarcinoma (LUAD), they were mainly associated with cell cycle, apoptosis, and RTK pathways (Figure 1A). In LUAD, lncRNAs predominantly exhibited negative correlations with the cell cycle and apoptosis pathways, whereas the RTK, Ras/MAPK, and PI3K/AKT pathways were primarily positively regulated (Figure 1B). The cell cycle pathway, which governs cell division and proliferation, plays a critical role in cancer therapy, as its dysregulation in tumor cells renders it a central target for therapeutic interventions.^[^ ^26^ ^]^ We observed that the lncRNA SFTA1P was negatively correlated with the cell cycle pathway score in LUAD (Figure 1C, Rs = −0.58, P = 3.6 × 10^−34^). Similarly, NAGPA‐AS1, displayed a negative correlation with the cell cycle pathway (Figure 1D, Rs = −0.57, P = 5.4 × 10^−32^), suggesting these lncRNAs may exert an inhibitory effect on the cell cycle, potentially suppressing tumor growth and progression in cancers with dysregulated cell cycle activity.

We used BRCA as an example to illustrate the detailed effects of lncRNAs on cancer signaling pathways due to its high heterogeneity and resistance to anti‐cancer therapies.^[^ ^27^ ^]^ In BRCA, lncRNAs predominantly positively regulate hormone ER and AR pathways while negatively regulating the cell cycle and apoptosis pathways (Figure S1A, Supporting Information). LncRNA AL078582.2 and the hormone ER pathway exhibited a strong and significant correlation (Figure S1B, Supporting Information, Rs = 0.75, P = 3 × 10^−157^). Estrogen receptor (ER) signaling plays a central role in driving ER‐positive breast carcinogenesis, and its inhibition remains the cornerstone of therapy for ER‐positive breast cancer, leading to substantial improvements in patient survival.^[^ ^28^ ^]^ Estrogen receptor α (ERα) is particularly critical in luminal breast cancer progression, promoting tumor growth through the activation of a hormone‐dependent transcriptional program.^[^ ^29^ ^]^ AL078582.2 is antisense to *ESR1*, the gene encoding ERα, suggesting that it may function by stabilizing *ESR1* mRNA, enhancing transcription, or promoting epigenetic activation to increase ESR1 expression. Further research is needed to clarify this mechanism. Another example is the association between lncRNA GATA3‐AS1 and hormone ER pathway (Figure S1C, Supporting Information, Rs = 0.64, P = 9.1 × 10^−103^). GATA3‐AS1, on the other hand, has been shown to promote tumor progression and immune evasion in triple‐negative breast cancer by destabilizing GATA3 while stabilizing PD‐L1.^[^ ^30^ ^]^ This suggests that downregulation of GATA3‐AS1 could potentially inhibit the hormone ER pathway in breast cancer, although further studies are required to elucidate the regulatory mechanism linking GATA3‐AS1 and ER signaling pathway.

Additionally, we performed a comprehensive PubMed search using keywords such as “lncRNA,” “cancer,” and “pathway,” which yielded a curated list of lncRNA‐pathway pairs supported by experimental evidence consistent with our findings (Table S2, Supporting Information).^[^ ^31^, ^32^, ^33^, ^34^, ^35^, ^36^, ^37^, ^38^, ^39^, ^40^, ^41^, ^42^, ^43^, ^44^, ^45^, ^46^, ^47^ ^]^ Taken together, our analysis offers a comprehensive and statistically robust overview of lncRNA‐mediated regulation of cancer‐related signaling pathways.

### 2.2 Associations between lncRNAs and Drug Responses

**Figure 2:**
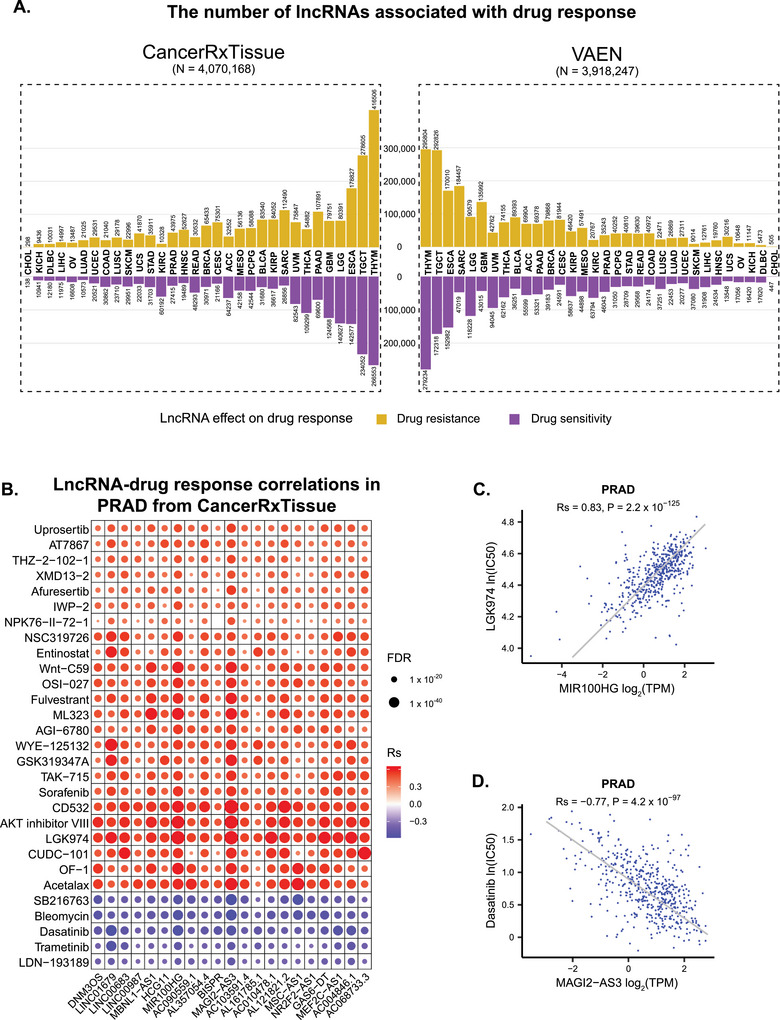
Associations between lncRNAs and imputed drug response from CancerRxTissue and VAEN. A) The number of lncRNAs associated with drug resistance (yellow) and drug sensitivity (purple) effects based on imputed drug responses from CancerRxTissue (left) and VAEN (right) across different cancer types. B) Top lncRNAs with the largest number of significantly associated imputed drug responses in prostate adenocarcinoma (PRAD). Dot color represents the correlation coefficient (Rs), and dot size corresponds to the FDR value. C) Positive correlation between MIR100HG and response to LGK974 in PRAD. D) Negative correlation between MAGI2‐AS3 and response to dasatinib in PRAD.

To investigate the effects of lncRNAs on drug responses, we performed Spearman's correlation analyses between lncRNA expression levels and imputed drug response values for 251 drugs or components from CancerRxTissue^[^ ^48^ ^]^ and VAEN,^[^ ^49^ ^]^ respectively, across tumor samples from 33 TCGA cancer types. A stringent threshold (|Rs| > 0.3 and FDR < 0.05) was applied to identify significant associations. In total, we identified 4 070 168 and 3 918 247 significant correlations between lncRNAs and imputed drug responses from CancerRxTissue and VAEN, respectively, encompassing both drug resistance and sensitivity effects (**Figures** **2**A, S2A,B, Table S3, Supporting Information). Prostate cancer is one of the most prevalent and clinically significant malignancies worldwide^[^ ^50^ ^]^ and is well‐known for its ability to develop resistance to standard therapies.^[^ ^51^ ^]^ Using prostate adenocarcinoma (PRAD) associations from CancerRxTissue as an example, the top lncRNAs associated with the greatest number of drugs exhibited overall positive correlations (Figure 2B). For instance, MIR100HG showed a strong positive correlation with drug LGK974, indicating a resistance effect of MIR100HG on LGK974 (Figure 2C, Rs = 0.83, P = 2.2 × 10^−125^). MIR100HG has been reported to function as a potential oncogene in various carcinomas.^[^ ^52^ ^]^ LGK974 is an orally bioavailable inhibitor that suppresses tumor growth in rodent xenograft models of pancreatic tumors.^[^ ^53^ ^]^ The observed resistance effect implies that inhibition of MIR100HG may enhance the therapeutic efficacy of LGK974 by mitigating resistance mechanisms. Another example is the negative correlation between MAGI2‐AS3 and the tyrosine kinase inhibitor dasatinib, indicating a sensitivity effect of MAGI2‐AS3 (Figure 2D, Rs = −0.77, P = 4.2 × 10^−97^). MAGI2‐AS3 functions as a tumor suppressor by suppressing the growth and migration of prostate cancer cells.^[^ ^54^ ^]^ Dasatinib has been demonstrated to inhibit prostate cancer growth and metastasis.^[^ ^55^, ^56^, ^57^ ^]^ The sensitivity effect of MAGI2‐AS3 on dasatinib indicated that its upregulation may potentiate the therapeutic response to dasatinib‐based treatments in prostate cancer.

### 2.3 Associations between lncRNAs and Immune Checkpoint Genes

**Figure 3:**
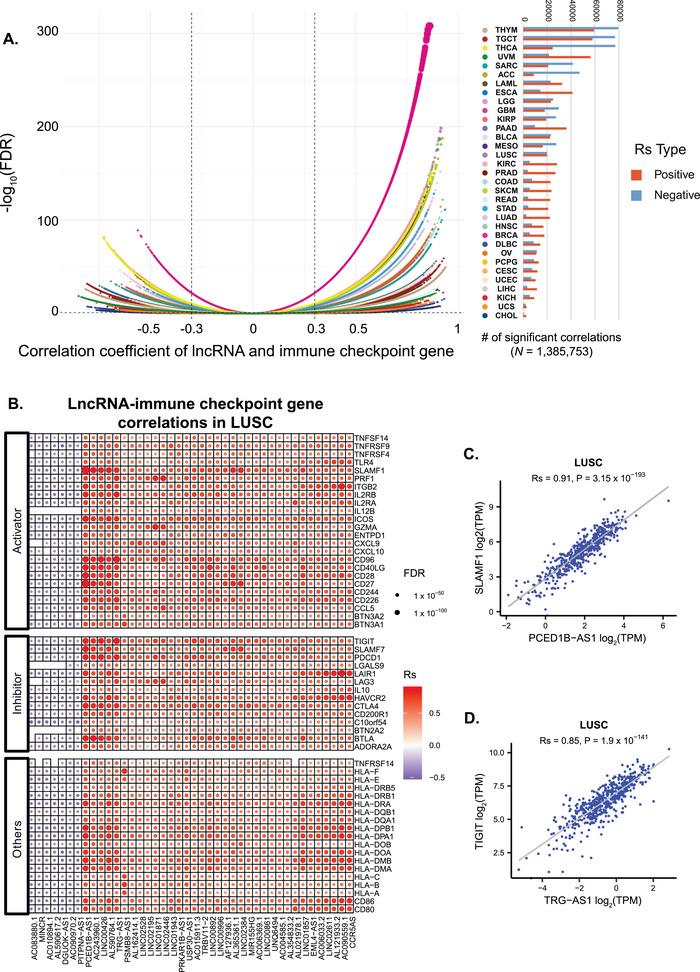
Associations between lncRNAs and immune checkpoint genes. A) Results of Spearman's correlation analysis (|Rs| > 0.3 and FDR < 0.05) (left) and the number of significant lncRNA‐immune checkpoint gene pairs across different cancer types (right). B) Top lncRNAs with the largest number of significantly associated immune checkpoint genes in lung squamous cell carcinoma (LUSC). Dot color represents the correlation coefficient (Rs), and dot size corresponds to the FDR value. C) Positive correlation between PCED1B‐AS1 and the immune checkpoint gene SLAMF1 in LUSC. D) Positive correlation between TRG‐AS1 and the immune checkpoint gene TIGIT in LUSC.

To explore the associations between lncRNAs and immune checkpoints, we performed Spearman's correlation analyses between lncRNA expression levels and 123 immune checkpoint genes identified in previous studies.^[^ ^58^, ^59^, ^60^ ^]^ A stringent threshold (|Rs| > 0.3 and FDR < 0.05) was applied to identify significant associations. In total, 1 385 753 significant associations were identified across 33 cancer types, ranging from 3030 in cholangiocarcinoma (CHOL) to 138 917 in THYM, with a median of 30 572 (**Figure** **3**A, Table S4, Supporting Information). Each immune checkpoint gene exhibited distinct lncRNA regulatory patterns across different cancer types (Figure S3, Supporting Information). For example, in lung squamous cell carcinoma (LUSC), the top lncRNAs correlated with the greatest number of immune checkpoint genes exhibited overall positive correlations, regardless of whether these genes function as activators, inhibitors, or had other modulatory roles (Figure 3B). The checkpoint gene SLAMF1 encodes a glycoprotein expressed on various immune cells, such as T cells, B cells, and NK cells, and plays a key role in immune regulation and intercellular communication.^[^ ^61^ ^]^ The strong positive correlation between PCED1B‐AS1 and SLAMF1 (Figure 3C, Rs = 0.91, P = 3.15 × 10^−193^) suggests that PCED1B‐AS1 may regulate SLAMF1 expression to modulate immune cell behavior. Another example is the positive correlation between TRG‐AS1 and immune checkpoint gene TIGIT (Figure 3D, Rs = 0.85, P = 1.9 × 10^−141^). TIGIT is an established immunotherapy target known to regulate NK cell effector function and metastasis.^[^ ^62^, ^63^, ^64^, ^65^, ^66^, ^67^, ^68^ ^]^ The observed correlation indicated that TRG‐AS1 may contribute to shaping the immune microenvironment through regulation of TIGIT expression. Targeting TRG‐AS1 may therefore enhance the efficacy of TIGIT blockade therapies and improve antitumor immune responses in LUSC. Collectively, these findings highlight the potential of lncRNA‐immune checkpoint interactions as promising avenues for developing lncRNA‐based cancer immunotherapies.

Additionally, we compiled a list of experimental studies from PubMed (Table S5, Supporting Information)^[^ ^69^, ^70^, ^71^, ^72^, ^73^, ^74^, ^75^, ^76^, ^77^ ^]^ using keywords such as “lncRNA,” “cancer,” and “immune checkpoint.” These studies, consistent with our findings, provide experimental support for the observed statistical associations.

### 2.4 Associations between lncRNAs and Immune Infiltration

**Figure 4:**
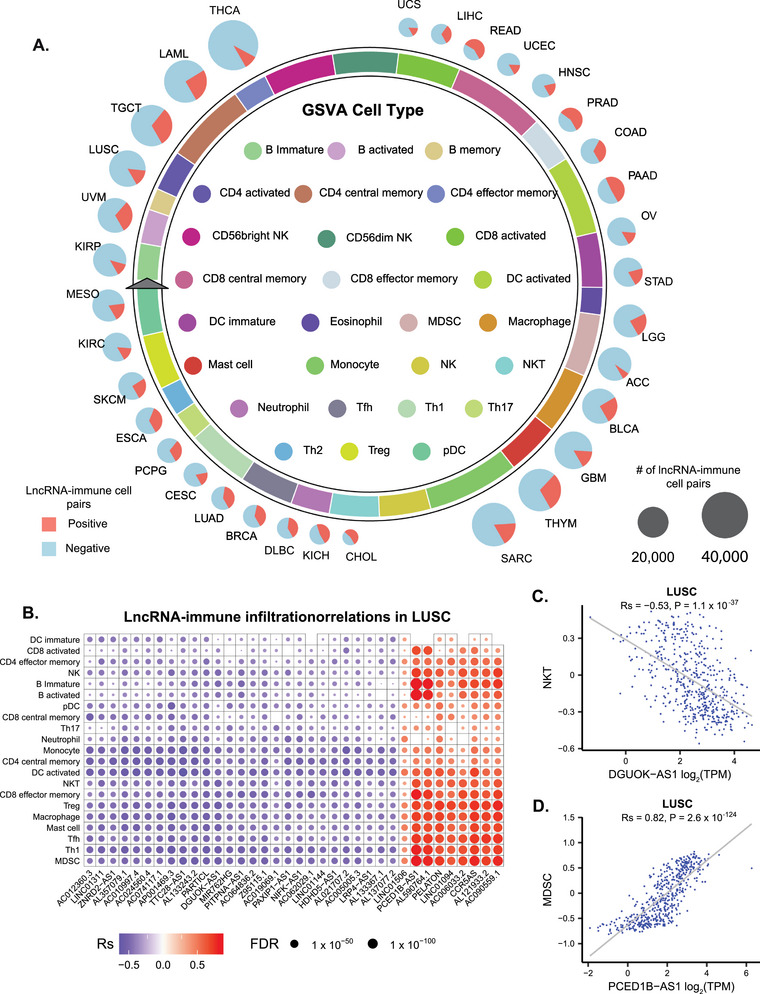
Associations between lncRNAs and immune infiltration. A) Proportion of lncRNAs correlated with immune cell abundances sourced from GSVA. GSVA contains 27 immune cell types (inner circle legend). The length of each immune cell segment on the circle represents the proportion of lncRNAs correlations across all cancer types. The outer circle cancer pie charts represent the proportion of positive (light red) and negative (light blue) correlations between lncRNAs and immune infiltration across all immune cell types. B) Top lncRNAs with the largest number of significant associations with immune infiltration in lung squamous cell carcinoma (LUSC). Dot color represents the correlation coefficient (Rs), and dot size corresponds to the FDR value. C) Negative correlation between DGUOK‐AS1 and NKT cells in LUSC. D) Negative correlation between PCED1B‐AS1 and MDSCs in LUSC.

To investigate the lncRNA effects on the tumor immune microenvironment (TIME), we performed Spearman's correlation analyses between lncRNA expression levels and immune infiltration data from eight different sources. A stringent threshold (|Rs| > 0.3 and FDR < 0.05) was applied to identify significant associations. In total, 1 860 214 lncRNA‐immune infiltration associations were identified, ranging from 56 170 associations across 22 cell types in CIBERSORT to 617 393 associations across 27 cell types in GSVA (Figure S4, Table S6, Supporting Information). Using GSVA‐based results as an example, the number of lncRNAs correlated with 27 immune cell types ranged from 9459 for B memory cells to 39 827 for CD8 central memory cells across all cancer types (**Figure** **4**A). The proportions of positive and negative correlations between lncRNAs and immune infiltration varied substantially among cancer types (Figure 4A).

We selected LUSC to illustrate the effects of lncRNAs on immune infiltration due to its extensive immune profiling data^[^ ^78^ ^]^ and high mortality.^[^ ^79^ ^]^ In LUSC, lncRNA exhibited predominantly negative correlations (85%) with immune cell abundance (Figure 4A), and 27 out of the 36 top lncRNAs with the largest number of significant associations exhibited overall negative correlations (Figure 4B). For example, we observed a negative correlation between DGUOK‐AS1 and NKT (Natural Killer T) cells (Figure 4C, Rs = −0.53, P = 1.1 × 10^−37^). DGUOK‐AS1 is upregulated in NSCLC, and its silencing inhibits cell proliferation, migration, invasion, and angiogenesis.^[^ ^80^ ^]^ NKT cells regulate immune response through interactions with dendritic cells, macrophages, and T cells.^[^ ^81^ ^]^ The negative correlation between DGUOK‐AS1 and NKT cell abundance indicated that DGUOK‐AS1 may suppress NKT cell infiltration. These findings suggest that targeting DGUOK‐AS1 could potentially enhance NKT cell‐mediated immune responses, offering a promising therapeutic strategy for LUSC. Another example is the positive correlation between PCED1B‐AS1 and MDSCs (Myeloid‐derived suppressor cells) (Figure 4D, Rs = 0.82, P = 2.6 × 10^−124^). PCED1B‐AS1 has been characterized as an oncogenic lncRNA in multiple cancers.^[^ ^82^, ^83^, ^84^ ^]^ MDSCs are a heterogeneous population of immune cells that suppress T‐cell and NK cell activity, promote immunosuppression, and facilitate tumor growth and metastasis within the tumor microenvironment.^[^ ^85^ ^]^

In BRCA, lncRNAs exhibited correlations across different cell types (Figure 4A), while the top lncRNAs with the largest number of significant associations exhibit overall positive correlations (Figure S4B, Supporting Information). Several of these lncRNAs were associated with key immune cell populations. For example, lncRNA PCED1B‐AS1 and TRG‐AS1 were positively correlated with CD8 effector memory cells (Figure S4C, Rs = 0.79, P = 6 × 10^−238^, & Figure S4D, Rs = 0.81, P = 1.8 × 10^−37^). CD8 effector memory T Cells represent a critical subset of memory T cells that mediate adaptive immune responses. High expression of a memory T cell signature has been associated with favorable prognosis and improved response to anti‐PD‐1 therapy in triple‐negative breast cancer.^[^ ^86^ ^]^ The positive correlations between these lncRNAs and immune cell abundance indicated that PCED1B‐AS1 and TRG‐AS1 may modulate the tumor immune microenvironment by promoting immune infiltration, thereby potentially enhancing the efficacy of immunotherapies.

In addition, to support the associations between lncRNAs and immune infiltration, we compiled a list of 11 experimental studies from PubMed using keywords such as “lncRNA,” “cancer,” and “immune” (Table S7, Supporting Information),^[^ ^87^, ^88^, ^89^, ^90^, ^91^, ^92^, ^93^, ^94^, ^95^, ^96^, ^97^ ^]^ which are consistent with our findings.

### 2.5 Associations between lncRNAs and the Efficacy/Toxicity of Immunotherapy

**Figure 5:**
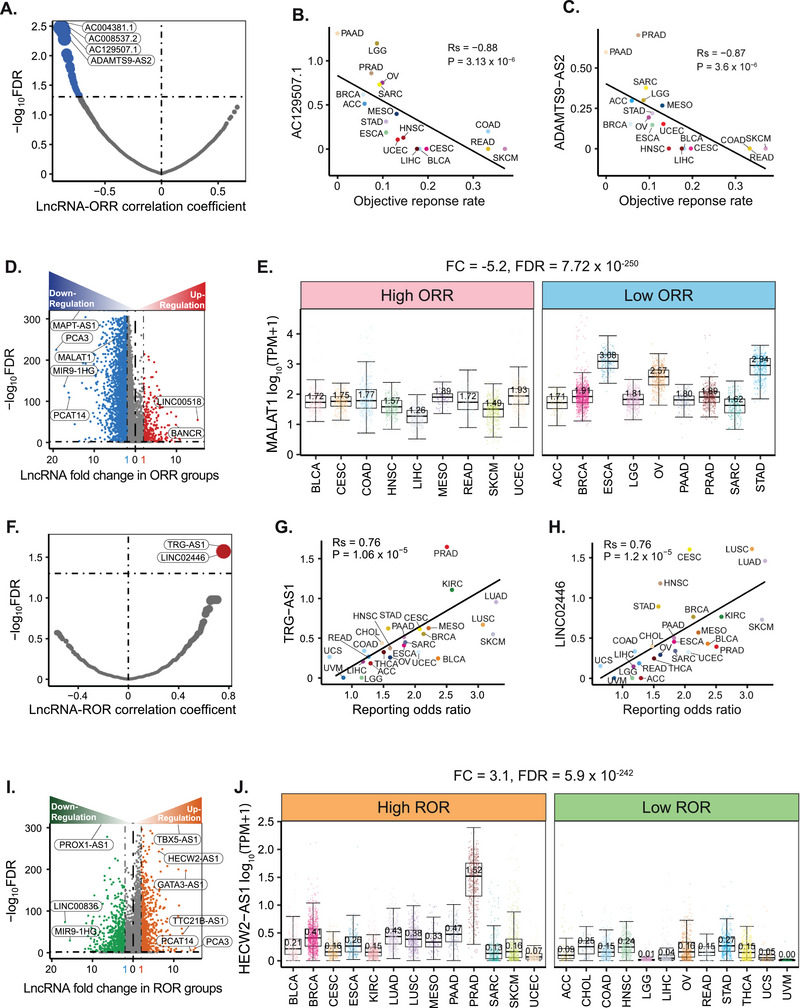
LncRNA impact on immunotherapy efficacy and toxicity. A) Spearman's correlation analysis between lncRNA expression levels and immunotherapy objective response rates (ORRs) (FDR < 0.05). B) Negative correlation between AC129507.1 and ORR. C) Negative correlation between ADAMTS9‐AS2 and ORR. D) Differential expression of lncRNAs between low‐ and high‐ORR cancer groups. E) MALAT1 was downregulated in the high ORR cancer group compared to the low ORR group. F) Spearman's correlation analysis between lncRNA expression levels and immune‐related adverse event (irAE) reporting odds ratios (RORs) (FDR < 0.05). G) Positive correlation between TRG‐AS1 and ROR. H) Positive correlation between LINC02446 and ROR. I) Differential expression of lncRNAs between low‐ and high‐ROR cancer groups. J) HECW2‐AS1 was upregulated in the high‐ROR cancer group compared to the low‐ROR group.

To examine the relationship of lncRNAs with immunotherapy outcomes, we performed an integrated analysis using immunotherapy objective response rates (ORRs)^[^ ^98^ ^]^ and immune‐related adverse event (irAE) reporting odds ratios (RORs)^[^ ^22^ ^]^ across different cancer types (Table S8, Supporting Information). We performed Spearman's correlation analyses between lncRNA expression levels and ORR to identify lncRNAs associated with immunotherapy efficacy. A stringent threshold (|Rs| > 0.3 and FDR < 0.05) was applied to identify significant associations. We identified 69 lncRNAs that were significantly negatively correlated with ORR (FDR < 0.05) (**Figure** **5**A), indicating that inhibition of these lncRNAs could potentially enhance the immunotherapy response. Among them, AC004381.1, AC008537.2, AC129507.1, and ADAMTS9‐AS2 showed the strongest negative correlations with ORR (Figure 5A). AC129507.1 has been identified as a risk lncRNA in stomach adenocarcinoma (STAD) patients,^[^ ^99^, ^100^ ^]^ while ADAMTS9‐AS2 functions as a tumor promoter in salivary adenoid cystic carcinoma^[^ ^101^ ^]^ and its expression has been significantly associated with immune infiltration in LUAD.^[^ ^102^ ^]^ The negative correlations observed between these lncRNAs and ORR (Figure 5B,C) suggested that suppressing their expression may improve immunotherapy efficacy, underscoring their potential as therapeutic targets for enhancing treatment outcomes.

To further identify lncRNAs associated with immunotherapy efficacy, we performed a differential expression analysis between cancer types with low and high ORRs. We considered |log_2_(fold change)| > 1 and FDR < 0.05 to be significant. In total, 269 lncRNAs were upregulated and 2342 lncRNAs were downregulated in the high ORR group compared to the low ORR group (Figure 5D). For example, MALAT1 was significantly downregulated in the high ORR group related to the low ORR group (Figure 5E, FC = −5.2, FDR = 7.72 × 10^−250^). MALAT1 has been implicated in promoting angiogenesis and immunosuppression across multiple cancer types.^[^ ^71^, ^103^ ^]^ Notably, combining a MALAT1 antisense oligonucleotide (ASO) with immune checkpoint blockade (ICB) therapy has been shown to enhance treatment responses in a preclinical model.^[^ ^104^ ^]^ Our findings are consistent with these studies, suggesting that MALAT1 downregulation may enhance anti‐cancer immune responses. We also identified several lncRNAs with significantly different expression levels between the high and low ORR groups that have not yet been studied, such as AL049838.1 and LIFR‐AS1 (Figure S5A, Supporting Information), highlighting the need for further investigation.

We performed Spearman's correlation analyses between lncRNA expression levels and irAE reporting odds ratios (ROR) to identify lncRNAs associated with irAE, using thresholds of |Rs| > 0.3 and FDR < 0.05 for significance (Figure 5F). We identified two lncRNAs, TRG‐AS1 (Figure 5G, Rs = 0.76, P = 1.06 × 10^−5^) and LINC02446 (Figure 5H, Rs = 0.76, P = 1.2 × 10^−5^), that were positively associated with irAE RORs, indicating the potential lncRNA biomarkers for irAE risk. Notably, expansion of ≥ 55 CD8 T‐cell clones has been observed prior to the onset of severe irAEs in patients treated with ipilimumab.^[^ ^23^ ^]^ Although the specific roles of TRG‐AS1 and LINC02446 in irAEs remain unexplored, our analyses revealed that both are significantly positively correlated with CD8 T cells infiltration across multiple cancer types (Figure S5B,C, Supporting Information). The strong positive correlations of TRG‐AS1 and LINC02446 with CD8 T cells suggest that these lncRNAs potentially modulate the irAEs induced by immune checkpoint blockade, representing potential biomarkers for predicting irAE risk and guiding immunotherapy strategies.

We further examined the differential expression of lncRNAs between cancer types with high and low irAE RORs, considering |log_2_(fold change)| > 1 and FDR < 0.05 as the significance criteria. In total, 687 lncRNAs were downregulated, while 689 lncRNAs were upregulated in the high ROR group compared to the low ROR group (Figure 5I). For example, HECW2‐AS1 was significantly upregulated in the high irAE ROR group relative to the low irAE ROR group (Figure 5J, FC = 3.1, FDR = 5.9 × 10^−242^). Although studies investigating lncRNAs in irAE remain limited, our analysis revealed that HECW2‐AS1 was significantly positively correlated with CD8 T cell infiltration across multiple cancer types from different immune infiltration sources (Figure S5D, Supporting Information). These findings suggest that HECW2‐AS1 may play a key role in modulating immune responses by influencing CD8 T cell activity, potentially contributing to the development of irAEs. Further investigation is warranted to elucidate its underlying mechanisms and evaluate its potential as a biomarker for irAE risk or as a therapeutic target.

### 2.6 A Comprehensive Data Portal, Pharmacogenomic and Immune Landscape of LncRNA

**Figure 6:**
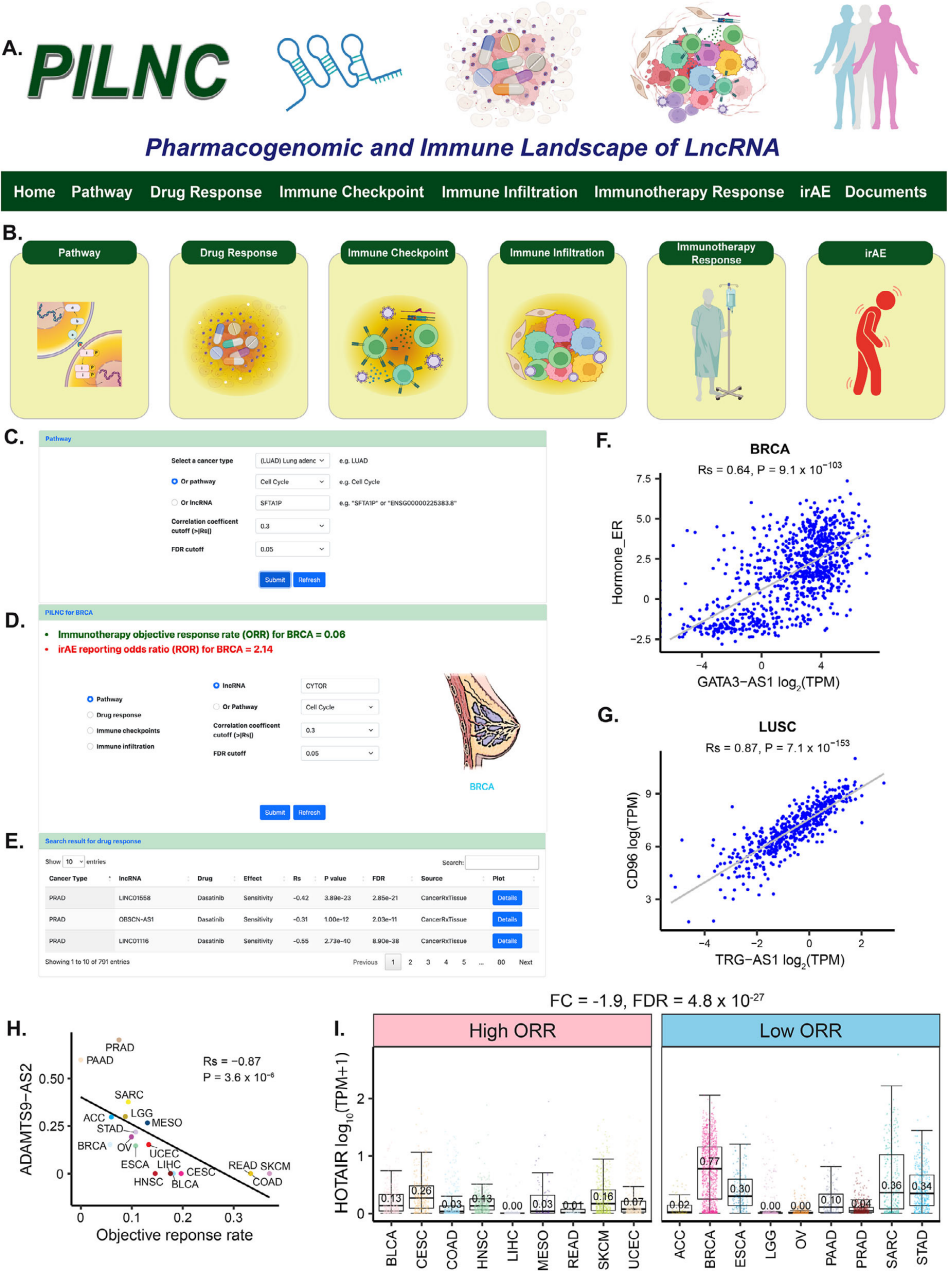
Content and interface of PILNC data portal. A) PILNC homepage and navigation bar. B) The six main functional modules of PILNC. C) Example of a search box in the Pathway module. D) Example of a search box in the cancer type‐specific search module. E) Example of a result table in the Drug Response module. F) Example of a scatter plot generated in the Pathway module. G) Example of a scatter plot generated in the Immune Checkpoint module. H) Example of a scatter plot from the Immunotherapy Response module using the Correlation method. I) Example of a box plot from the Immunotherapy Response module using the Differential expression method.

To enhance the visualization, searchability, and accessibility of our findings for the research community, we developed a user‐friendly data portal, PILNC (https://hanlaboratory.com/PILNC) (**Figure** **6**A). PILNC comprises six primary modules: Pathway, Drug Response, Immune Checkpoint, Immune Infiltration, Immunotherapy Response, and irAE (Figure 6B). Users can query data by selecting parameters such as cancer type, lncRNA, pathway, drug or component, immune checkpoint, or immune cell type and can define statistical cutoffs for correlation coefficient (Rs), fold change (|log_2_(FC)|), and FDR significance (Figure 6C). For example, in the **Pathway** module, users can explore lncRNA‐pathway associations by selecting a cancer type (e.g., LUAD), choosing a pathway (e.g., Cell Cycle), or entering a specific lncRNA name or ensemble ID (e.g., SFTA1P, or ENSG00000225383.8). In the **Drug Response** module, users can examine lncRNA‐drug response associations by specifying a cancer type (e.g., PRAD), selecting a drug response source (e.g., CancerRxTissue), choosing a drug or component (e.g., Dasatinib), or entering a lncRNA name or ensemble ID (e.g., MAGI2‐AS3, or ENSG00000234456.8). In the **Immune Checkpoint** module, users can search for lncRNA‐immune checkpoint associations by selecting a cancer type (e.g., LUSC), selecting from 123 immune checkpoint genes (e.g., SLAMF1), or entering a lncRNA name or ensemble ID (e.g., PCED1B‐AS1, or ENSG00000247774.7). Similarly, in the **Immune Infiltration** module, users can investigate lncRNA‐immune cell associations by selecting a cancer type (e.g., LUSC), choosing an immune cell estimation source (e.g., GSVA), selecting a specific immune cell type (e.g., MDSC), or entering a lncRNA name or ensemble ID (e.g., PCED1B‐AS1, or ENSG00000247774.7). In the **Immunotherapy Response** module, users can identify lncRNAs associated with immunotherapy response using two approaches: (1) correlations between lncRNA expression and ORR, or (2) differential expression analyses between low‐ and high‐ ORR cancer groups. Likewise, in the **irAE** module, users can identify lncRNAs associated irAE through (1) correlations between lncRNA expression and ROR, or (2) differential expression analyses between low‐ and high‐ ROR cancer groups. Additionally, “**Browse by cancer types**” section at the bottom of the main page allows users to click on a specific cancer type (e.g., BRCA) to view corresponding ORR and ROR values and to access related results across the Pathway, Drug Response, Immune Checkpoint and Immune Infiltration modules (Figure 6D). Search results in each module are presented in a tabulated format, enabling users to systematically view and filter relevant information (Figure 6E). For each entry, a “Details” button allows users to generate specific visualizations, such as scatter plots, within different modules (Figure 6F,G). Furthermore, users can visualize data using correlation‐based scatter plots (Figure 6H) and box plots derived from differential expression analyses within the Immunotherapy Response and irAE modules (Figure 6I).

For example, if users wish to investigate the impact of the lncRNA PCED1B‐AS1 on STAD and its association with immunotherapy outcomes, they can navigate to the “**Browse by cancer types**” module and click the “**STAD**” icon. The **ORR** and **ROR** values will appear in the upper‐left corner of the search box. Users can then select one of the modules, “**Pathway,**” “**Drug Response,**” “**Immune Checkpoint,**” or “**Immune Infiltration,**” and enter either “PCED1B‐AS1” or its ensemble ID “ENSG00000247774.7” into the “lncRNA” search field (Figure S6A, Supporting Information). The corresponding results will be displayed in a tabulated table (Figure S6B, Supporting Information), and by clicking “Detail,” users can generate scatter plots to visualize specific associations (Figure S6C,D, Supporting Information). In addition to viewing ORR and ROR values in the cancer‐specific window, users can further explore the relationships between lncRNAs and immunotherapy outcomes through the Immunotherapy Response and irAE modules (Figure S6E, Supporting Information). After obtaining results from either the “Correlation” or “Differential Expression” analysis, users can enter a lncRNA name (i.e., PCED1B‐AS1) in the upper‐right corner of the result table to filter for their lncRNA of interest (Figure S6F, Supporting Information). By clicking the “Detail” button, users can access the corresponding visualization plots (Figure S6G,H, Supporting Information).

The PILNC data portal allows users to explore the multi‐dimensional functions of key lncRNAs across multiple modules. For example, in BRCA, AC092718.4 showed a negative correlation with the ER signaling pathway score (Rs = −0.38, P = 2.3 × 10^−30^), suggesting its potential inhibitory role in estrogen receptor‐mediated transcriptional activity. AC092718.4 also exhibited significant sensitivity to Tamoxifen (Rs = −0.42, P = 3.5 × 10^−46^), a drug targeting ESR1 in the hormone ER pathway, further supporting its involvement in modulating the response to ER‐targeted therapies.

MEG3 showed inhibitory effects on the cell cycle pathway in BRCA (Rs = −0.3, P = 1.5 × 10^−18^), suggesting its potential tumor‐suppressive role through regulation of cell cycle activity. MEG3 also displayed significant sensitivity to CCT007093 (Rs = −0.35, P = 8.6 × 10^−31^), a drug targeting the cell cycle pathway, further supporting its involvement in enhancing drug responsiveness. With respect to immune features, MEG3 was positively associated with several immune cell populations, including CD4 T cells (Rs = 0.46, P = 3.5 × 10^−55^), and NK cells (Rs = 0.34, P = 1.1 × 10^−29^), highlighting its potential involvement in modulating immune infiltration. Notably, MEG3 expression was negatively correlated with the immunotherapy objective response rate (ORR) (Rs = −0.7, P = 4.7 × 10^−2^), suggesting that MEG3 may serve as a potential biomarker for predicting immunotherapy response.

Additionally, the PILNC data portal allows users to investigate lncRNA effects across multiple cancer types within each module by selecting “All cancer types.” For example, SLC16A1‐AS1 exhibited significant sensitivity to Palbociclib in BRCA (Rs = −0.37, P = 8.7 × 10^−36^), as well as in CESC (Rs = −0.4, P = 2.2 × 10^−11^), THYM (Rs = −0.44, P = 2.2 × 10^−6^), and UVM (Rs = −0.37, P = 7.4 × 10^−3^), suggesting a potentially sensitivity to Palbociclib across multiple cancer types. For immune infiltration, SLC16A1‐AS1 showed strong positive associations with CD8 T cells infiltration in several cancers, including BLCA (Rs = 0.43, P = 4.2 × 10^−18^), LUAD (Rs = 0.33, P = 9.2 × 10^−13^), and PAAD (Rs = 0.47, P = 2.9 × 10^−9^), suggesting its association with CD8 T cell‐enriched tumor microenvironment.

Similarly, lncRNA AC090152.1 exhibited strong drug sensitivity to Epirubicin across multiple cancer types, including ACC (Rs = −0.33, P = 4 × 10^−2^), BRCA (Rs = −0.51, P = 5.6 × 10^−72^), COAD (Rs = −0. 4, P = 3.8 × 10^−17^), LGG (Rs = −0.37, P = 4.6 × 10^−17^), LIHC (Rs = −0.36, P = 1.4 × 10^−10^), PAAD (Rs = −0.34, P = 3.5 × 10^−5^), and THYM (Rs = −0.82, P = 5.7 × 10^−28^), indicating its potential involvement in modulating Epirubicin responsiveness across cancers. Regarding immune features, AC090152.1 showed positive correlations to immune checkpoint gene PDCD1 across several cancer types, including BLCA (Rs = 0.46, P = 5.2 × 10^−21^), BRCA (Rs = 0.59, P = 3.1 × 10^−99^), CESC (Rs = 0.36, P = 1 × 10^−8^), KIRC (Rs = 0.56, P = 2.1 × 10^−43^), LIHC (Rs = 0.45, P = 6.4 × 10^−18^), MESO (Rs = 0.39, P = 6.9 × 10^−3^), SKCM (Rs = 0.44, P = 1.5 × 10^−21^), THCA (Rs = 0.36, P = 7.9 × 10^−16^), and THYM (Rs = 0.61, P = 9.8 × 10^−12^), These consistent positive associations suggest that AC090152.1 may contribute to the regulation of PDCD1 expression, implicating it in the response to immune checkpoint blockade therapy.

## 3 Discussion

As oncology research advances, the integration of lncRNAs into targeted and immunotherapeutic strategies presents promising opportunities to enhance the precision and efficacy of cancer treatments.^[^ ^105^ ^]^ In this study, we systematically investigated the impact of lncRNAs on cancer signaling pathways utilizing pathway activity scores derived from RPPA500 protein expression data from TCPA. We further analyzed lncRNA associations with drug responses by leveraging imputed drug response values from two large‐scale pharmacogenomic databases, CancerRxTissue and VAEN. Additionally, we characterized the immune landscape of lncRNAs by examining their associations with immune checkpoint gene expression and immune infiltration profiles from eight different data sources. We also explored the relationships between lncRNAs and real‐world pharmacovigilance data, identifying lncRNAs associated with both immunotherapy efficacy and irAEs. To ensure broad accessibility and facilitate exploration of these findings, we developed a user‐friendly data portal, PILNC (https://hanlaboratory.com/PILNC), which provides researchers with interactive tools to analyze and visualize lncRNA‐associated features across cancers. Collectively, this integrated multi‐omics approach establishes a comprehensive framework for uncovering lncRNA‐mediated mechanisms and offers valuable insights into potential therapeutic targets for precision oncology.

We identified 53 173 lncRNA‐pathway associations across ten cancer signaling pathways in 33 cancer types, highlighting the diverse and critical functions of lncRNAs in cancer progression. In addition, we detected 391 8247 and 4 070 168 lncRNA‐drug response associations from the CancerRxTissue and VAEN imputed drug response sources, respectively, across 33 cancer types, revealing the extensive influence of lncRNAs on therapeutic sensitivity and resistance. These associations provide valuable insights into how lncRNAs may modulate the efficacy of anticancer agents, offering potential avenues for developing personalized treatment strategies. Furthermore, we uncovered 1 385 753 lncRNA‐immune checkpoint gene associations and 1 860 214 lncRNA‐immune infiltration associations, emphasizing the substantial impact of lncRNAs on ICPs and the TIME. Together, these findings highlight the pivotal role of lncRNAs in regulating immune checkpoint expression and shaping immune infiltration, underlining their potential as modulators of immune responses and as promising therapeutic targets for enhancing immunotherapy efficacy.

We systematically investigated the relationship between lncRNAs and immunotherapy outcomes using real‐world pharmacovigilance data, including immunotherapy objective response rate (ORR) and immune‐related adverse event (irAE) reporting odds ratio (ROR). This analysis identified a distinct set of lncRNAs significantly associated with either ORR or irAE ROR. Combined with lncRNAs linked to immune checkpoints and immune infiltration, these findings provide a comprehensive view of the immunoregulatory roles of lncRNAs and highlight promising candidates for advancing cancer immunotherapy.

The immense value of rigorous analysis using large‐scale data highlights the transformative potential of computational approaches in advancing our understanding of cancer biology.^[^ ^106^, ^107^, ^108^, ^109^, ^110^, ^111^, ^112^ ^]^ In this study, we integrated multi‐dimensional results to develop PILNC, a user‐friendly and a comprehensive data portal designed to elucidate the intricate pharmacogenomic and immune landscape of lncRNAs. Our integrative analyses revealed that several lncRNAs identified in PILNC, including MEG3 and SLC16A1‐AS1, possess translational potential as candidate biomarkers and therapeutic targets. By integrating pathway activity, pharmacogenomic sensitivity, immune profiling, and immunotherapy outcomes, PILNC provides a comprehensive framework for prioritizing lncRNAs with multi‐layered functional relevance and clinical utility. Future experimental validation will be essential to confirm these computationally predicted targets and elucidate their mechanistic roles in cancer progression and treatment response. PILNC serves as a valuable resource for investigating the biological and clinical significance of lncRNAs in the context of targeted therapy and immunotherapy. To ensure its continued relevance, we are committed to regularly updating PILNC with emerging datasets, maintaining it as a dynamic and evolving platform that supports ongoing discoveries in cancer pharmacogenomics and immunology.

This study emphasizes the pivotal role of lncRNAs in driving cancer progression, shaping the tumor immune microenvironment, and influencing therapeutic responses. It lays a solid foundation for future investigation into the functional and clinical applications of lncRNAs, highlighting their potential to advance the precision and efficacy of targeted therapies and immunotherapies, and ultimately paving the way for more effective and personalized cancer treatments.

## 4 Experimental Section

### Data from The Cancer Genome Atlas

Bulk RNA‐seq lncRNA expression data (TPM) were obtained using the “TCGAbiolinks” R package.^[^ ^113^ ^]^ LncRNAs with an average TPM ≥ 0.3 across samples within each cancer type were retained and defined as detectable lncRNAs in this analysis.^[^ ^114^ ^]^ The final dataset included 12 568 lncRNAs from 11 275 samples across 33 cancer types, ranging from 3261 in UVM to 7488 in LAML (Table S9, Supporting Information). mRNA expression profiles from the same 33 tumor types were downloaded from the TCGA data portal (https://portal.gdc.cancer.gov/). Cancer signaling pathway scores were calculated using the RPPA500 dataset from The Cancer Proteome Atlas (TCPA)^[^ ^25^ ^]^ following the method described previously.^[^ ^115^ ^]^ Briefly, pathway scores were derived by summing the relative protein levels of all positive regulatory components and subtracting the sum of negative regulatory components within the pathway.^[^ ^115^ ^]^

### Drug Response Data

For pharmacogenomic analysis, this work utilized Half‐maximal inhibitory concentration (IC_50_) values from two reliable sources: CancerRxTissue (https://manticore.niehs.nih.gov/cancerRxTissue/)^[^ ^48^ ^]^ and VAEN (https://github.com/bsml320/VAEN/),^[^ ^49^ ^]^ each of which has been widely used in our previous studies.^[^ ^116^, ^117^ ^]^ To ensure consistency and accurate interpretation of drug information, drug nomenclature was standardized using the Genomics of Drug Sensitivity in Cancer (GDSC) database.

### Immune Infiltration Data

For immune infiltration analysis, this work integrated data from TIMER2.0^[^ ^118^ ^]^ which offers robust estimates of immune infiltration levels in TCGA tumor samples using six advanced algorithms:^[^ ^119^ ^]^ TIMER,^[^ ^120^ ^]^ xCell,^[^ ^121^ ^]^ MCP‐counter,^[^ ^122^ ^]^ CIBERSORT,^[^ ^123^ ^]^ EPIC,^[^ ^124^ ^]^ and quanTIseq.^[^ ^125^ ^]^ Additionally, immune cell abundance from the ImmuCellAI^[^ ^126^ ^]^ and GSVA^[^ ^112^ ^]^ were also incorporated. In total, 67 unique cell types/statuses were included in the analysis, ensuring comprehensive coverage of the tumor immune microenvironment. This multi‐source, multi‐algorithm integration strengthens the robustness and reliability of immune infiltration estimates in our study.

We obtained immunotherapy objective response rates (ORRs) from a previous study that analyzed data from 27 tumor types.^[^ ^98^ ^]^ Briefly, ORR was calculated using the formula:

(1) $$10.8\times \mathrm{loge}\left(\times \right)-0.7$$

where “X” represents the number of coding somatic mutations per megabase of DNA.^[^ ^98^ ^]^

Additionally, immune‐related adverse event (irAE) reporting odds ratios (RORs) were derived from our previous study.^[^ ^22^ ^]^ Briefly, irAE RORs were calculated by comparing the proportion of reporting irAEs for anti‐PD‐1/PD‐L1 agents with the proportion of reporting irAEs for all other drugs in the database.^[^ ^127^ ^]^

### Statistical Analysis

To identify associations between lncRNAs and pathway scores, drug responses, immune checkpoint gene expression, immune infiltration, and immunotherapy outcomes, we employed Spearman's correlation analysis, a non‐parametric method well suited for capturing monotonic relationships in noisy biological data. This approach provides robust performance across diverse data types. A stringent threshold (|Rs| > 0.3 and FDR < 0.05) was applied to define statistically significant associations.

For differential expression analysis, we employed the Wilcox rank‐sum test, a non‐parametric method widely recognized for its robustness in detecting differences between groups. To enhance reliability, only lncRNAs meeting specific inclusion criteria were analyzed: each comparison group (high/low ORR or high/low ROR) had to include at least three cancer types with detectable expression. LncRNAs with |log_2_(fold change)| > 1 and FDR < 0.05 were considered significantly differentially expressed.

### PILNC Database Development

The PILNC portal was developed using established frameworks, including the Python Flask framework^[^ ^128^ ^]^ and Bootstrap (https://getbootstrap.com/), to ensure a robust and user‐friendly platform for accessing and analyzing the lncRNA data. The web application is hosted on the Apache HTTP Server and deployed in the Jetstream2 cloud platform,^[^ ^129^, ^130^ ^]^ ensuring scalability and stability. The PILNC website is freely available online (https://hanlaboratory.com/PILNC/). Regular updates will be implemented to ensure data accuracy and relevance, further supporting the reliability of PILNC as a research resource.

## Conflict of Interest

The authors declare no conflict of interest.

## Author Contributions

R.W. and M.L. contributed equally to this work. L.H. conceived, designed, and supervised the project. R.W. and M.L. performed the research. R W., M.L., Y.L., J.Y., Y.C., C.C., and L.M. performed the data analyses. R.W., M.L., and Y.L. constructed the data portal. R.W., J.W., S.D., Y.Z., W.L., B.L., L.Y., C.L., L.D., and L.H. interpreted the results. R.W., M.L., LD., and L.H. wrote the manuscript. The authors read and approved the final manuscript.

## Supporting information



Supporting Information

Supporting Information

Supporting Information

## Data Availability

The data that support the findings of this study are openly available in PILNC data portal at https://hanlaboratory.com/PILNC/. These data were derived from the following resources available in the public domain: The Cancer Genome Atlas (TCGA), https://portal.gdc.cancer.gov/; The Cancer Proteome Atlas (TCPA), https://tcpa.drbioright.org/rppa500/main.html; CancerRxTissue, https://manticore.niehs.nih.gov/cancerRxTissue/; VAEN, https://github.com/bsml320/VAEN/; TIMER2.0, https://compbio.cn/timer2/.
