# Supplementary material for: Functional, Pharmacogenomic, and Immune Landscapes of Long Non‐Coding RNAs in Cancer
Source: Adv Sci (Weinh). 2025 Nov 21;13(6):e13414. doi: 10.1002/advs.202513414 (PMC12866694; doi:10.1002/advs.202513414)
Supplement: Supplementary file 1 — Supporting Information [file ADVS-13-e13414-s002.docx]

**Inventory of Supplementary Information**

**Supplementary Figure 1. LncRNA-cancer signaling pathway associations in breast invasive carcinoma (BRCA).** **A**, Top cancer signaling pathways associated with the largest number of lncRNAs in BRCA. LncRNAs and pathways are represented by dots on the outer and inner circles, respectively. The size of each dot reflects the number of correlations associated with the corresponding lncRNAs or pathways. Linkage lines between pathways and lncRNAs denote significant correlations, with line colors representing the correlation coefficient and line widths indicating the FDR value. **B**, Positive correlation between AL078582.2 and the hormone ER pathway score in BRCA. **C**, Positive correlation between GATA3-AS1 and the hormone ER pathway score in BRCA.

**Supplementary Figure 2. Top drugs associated with the largest number of lncRNAs across cancer types**. **A.** Results from **CancerRxTissue. B.** Results from **VAEN.** Yellow indicates lncRNAs with drug resistance effects, and purple indicates lncRNAs with drug sensitivity effects. The size of each pie represents the number of lncRNA associated with imputed drug responses.

**Supplementary Figure 3. Top immune checkpoint genes associated with the largest number of lncRNAs across cancer types.** Red indicates positive correlations, and blue indicates negative correlations. The size of pie represents the number of lncRNAs associated with each immune checkpoint gene.

**Supplementary Figure 4. Associations between lncRNAs and immune infiltration. A,** Number of lncRNA-immune infiltration associations from eight sources across different cancer types. **B,** Top immune cells associated with the largest number of lncRNAs from GSVA in breast invasive carcinoma (BRCA). Dot color represents the correlation coefficient (Rs), and dot size corresponds to the FDR value. **C**, Positive correlation between PCED1B-AS1 and CD8 effector memory T cells. **D**, Positive correlation between TRG-AS1 and CD8 effector memory T cells.

**Supplementary Figure 5. LncRNA impact on immunotherapy efficacy and toxicity. A,** AL049838.1 and LIFR-AS1were downregulated in the high immunotherapy objective response rate (ORR) group compared to the low ORR group. **B**, Positive correlation between TRG-AS1 and CD8 T cells across multiple cancer types from different sources. **C**, Positive correlation between LINC02446 and CD8 T cells in multiple cancer types from different sources. **D**, Positive correlation between HECW2-AS1 and CD8 T cells in multiple cancer types from different sources.

**Supplementary Figure 6. Investigation of lncRNA PCED1B-AS1 effects on** **stomach adenocarcinoma (STAD) and its immunotherapy outcomes in PILNC data portal. A,** Search box in the STAD-specific search module. **B**, Result table in the STAD-specific search module. **C**, Scatter plot showing the correlation between PCED1B-AS1 and CD4 T cells from the Immune Infiltration module. **D**, Scatter plot showing correlation between PCED1B-AS1 and the immune checkpoint gene ADORA2A from the Immune Checkpoint module. **E**, Search box in the irAE module. **F**, Filtered result for PCED1B-AS1 from the irAE module result table. **G**, Scatter plot showing the correlation between PCED1B-AS1 and ROR. **H**, Box plot showing PCED1B-AS1 fold change between low- and high-ROR cancer groups.

**Supplementary Table 1. Summary of the number of associations between lncRNAs and cancer signaling pathways.**

**Supplementary Table 2. Experimental studies supporting lncRNA-pathway associations.**

**Supplementary Table 3. Summary of the number of associations between lncRNAs and imputed drug response.**

**Supplementary Table 4. Summary of the number of associations between lncRNAs and immune checkpoint genes.**

**Supplementary Table 5. Experimental studies supporting lncRNA-immune checkpoint gene associations.**

**Supplementary Table 6. Summary of number of associations between lncRNAs and immune cell infiltrations.**

**Supplementary Table 7. Experimental studies supporting lncRNA-immune infiltration associations.**

**Supplementary Table 8. Immunotherapy objective response rates (ORRs) and immune-related adverse event (irAE) reporting odds ratios (RORs) available.**

**Supplementary Table 9. Summary of detectable lncRNAs and sample numbers from 33 cancer types.**
