# Supplementary material for: Functional, Pharmacogenomic, and Immune Landscapes of Long Non‐Coding RNAs in Cancer
Source: Adv Sci (Weinh). 2025 Nov 21;13(6):e13414. doi: 10.1002/advs.202513414 (PMC12866694; doi:10.1002/advs.202513414)

Supplementary Figure 1.

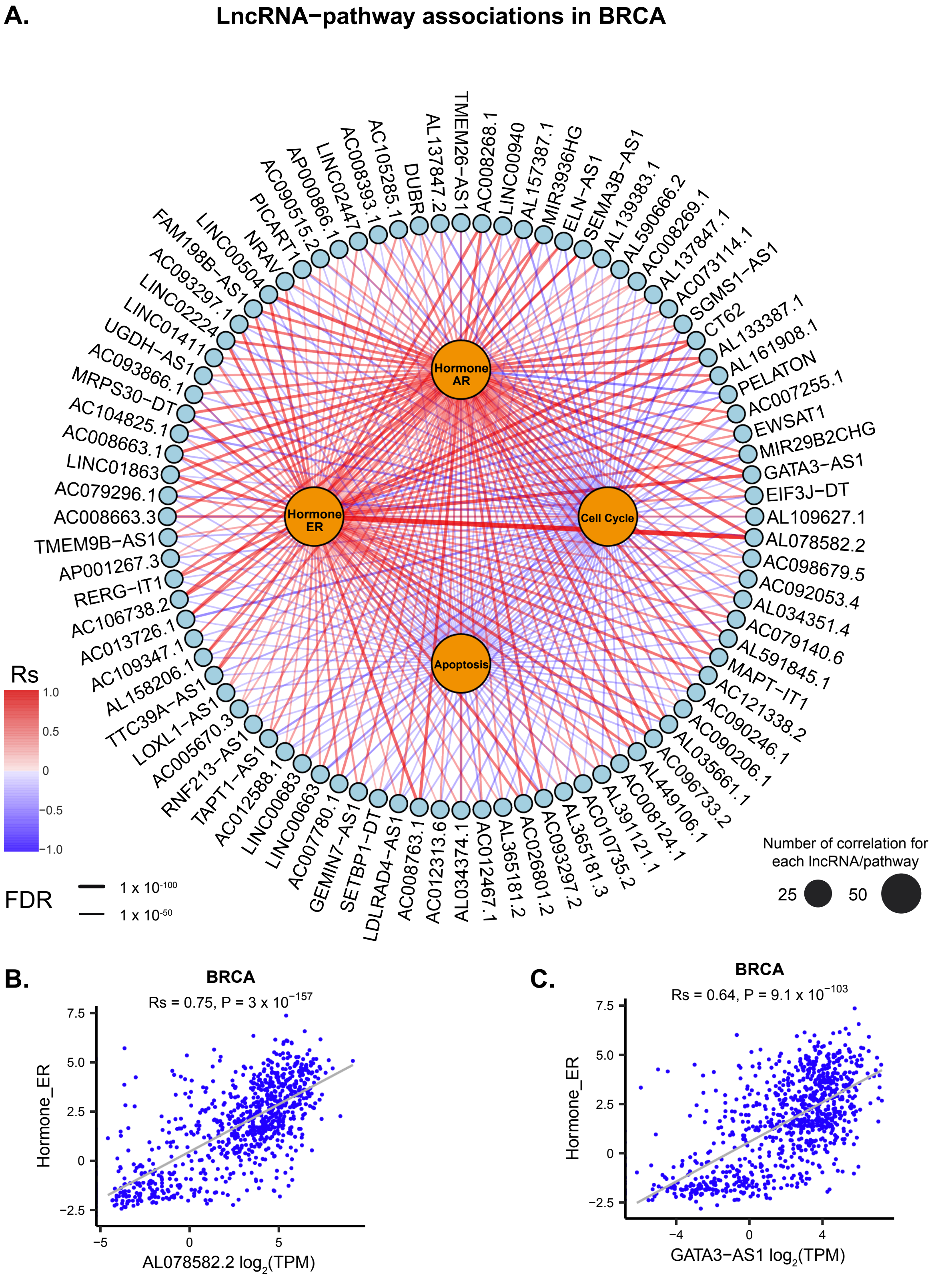

Supplementary Figure 2.

A. **Top drugs from CancerRxTissue associated with lncRNAs in different cancer types**

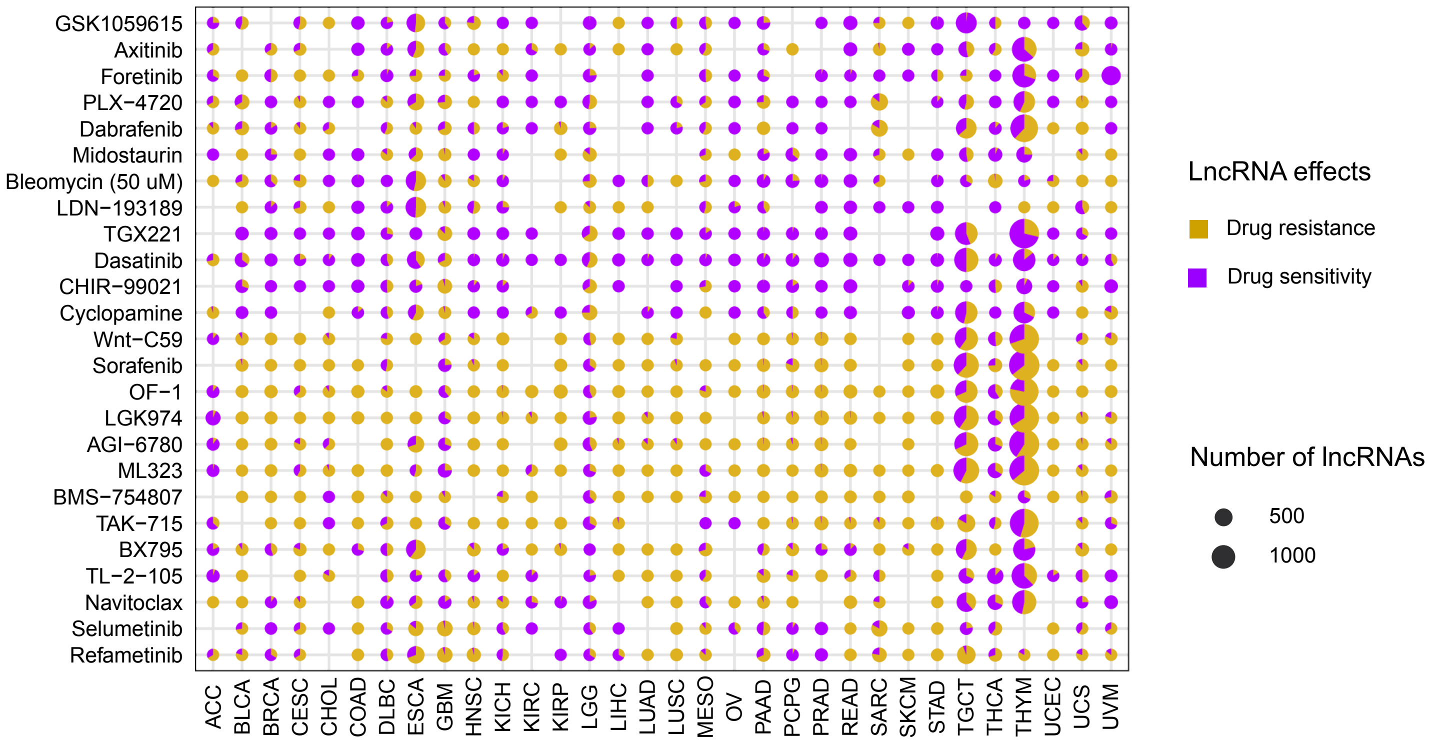

B. **Top drugs from VAEN associated with lncRNAs in different cancer types**

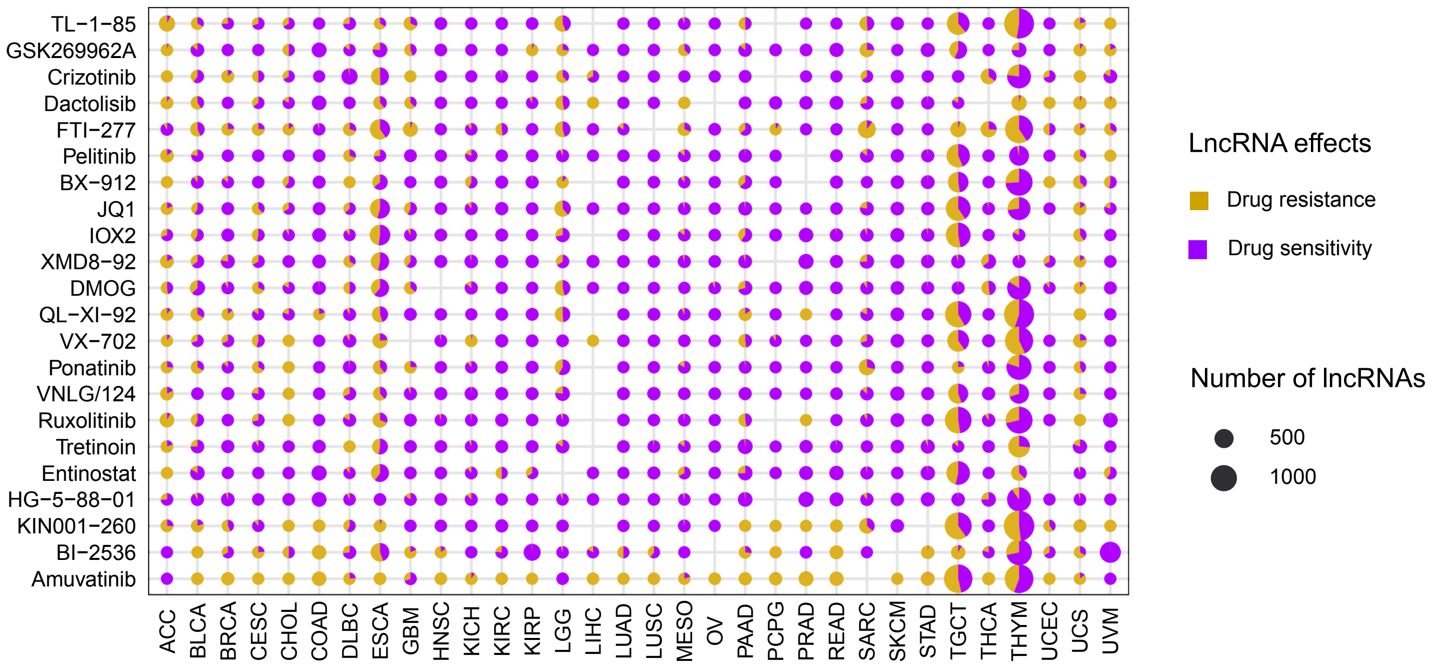

Supplementary Figure 3.

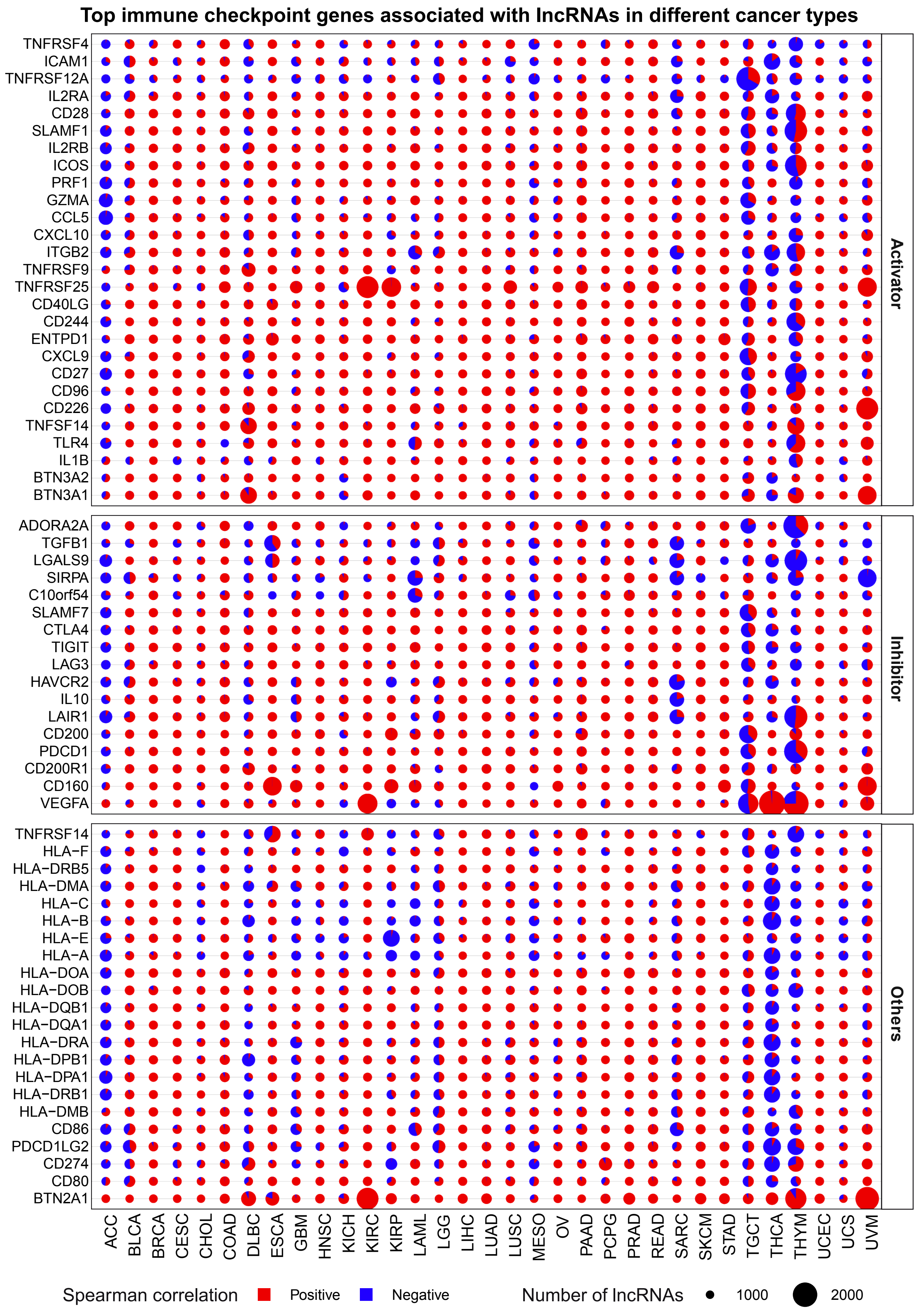

Supplementary Figure 4.

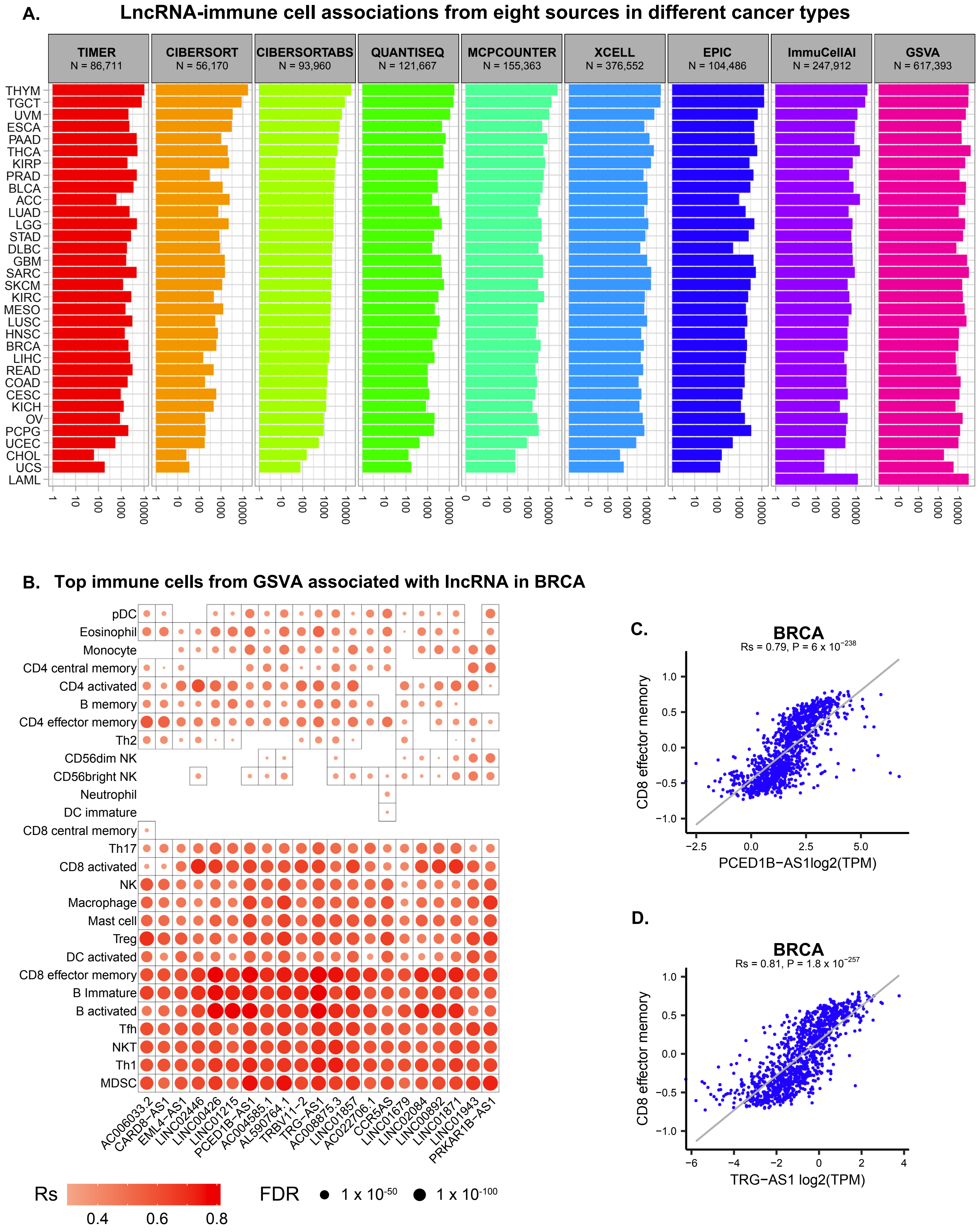

Supplementary Figure 5.

A.

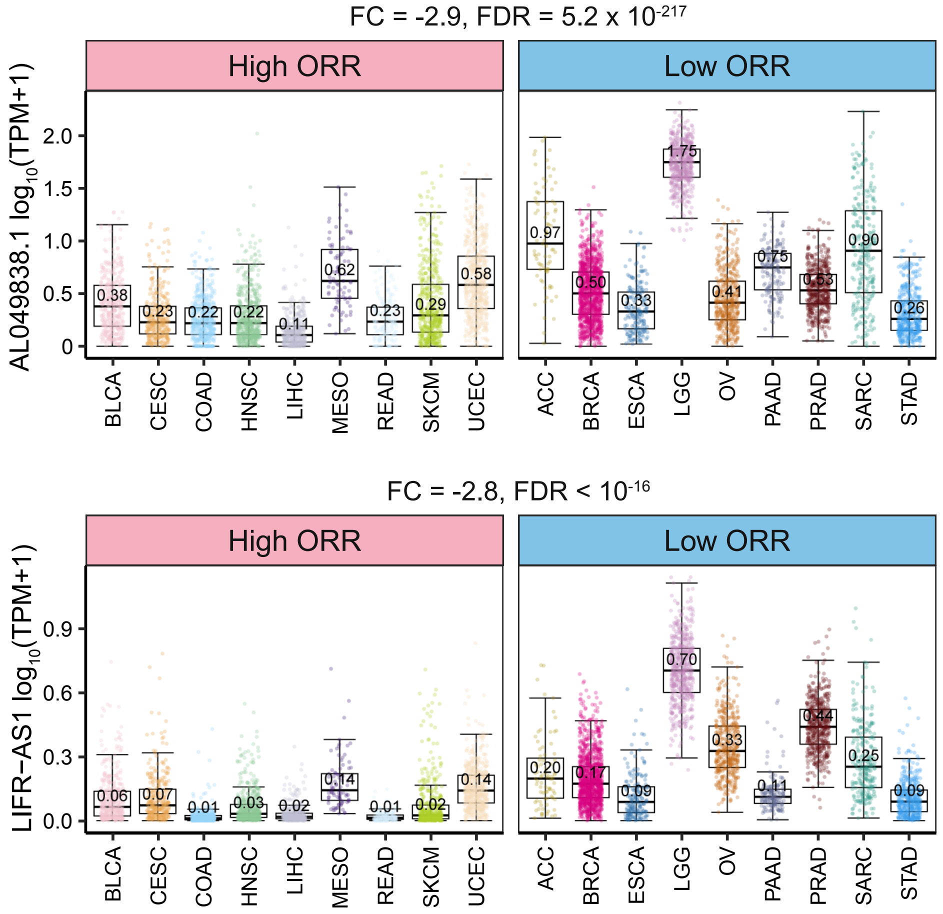

B. **LncRNA TRG-AS1 positively correlated with CD8 T cells in multiple cancers**

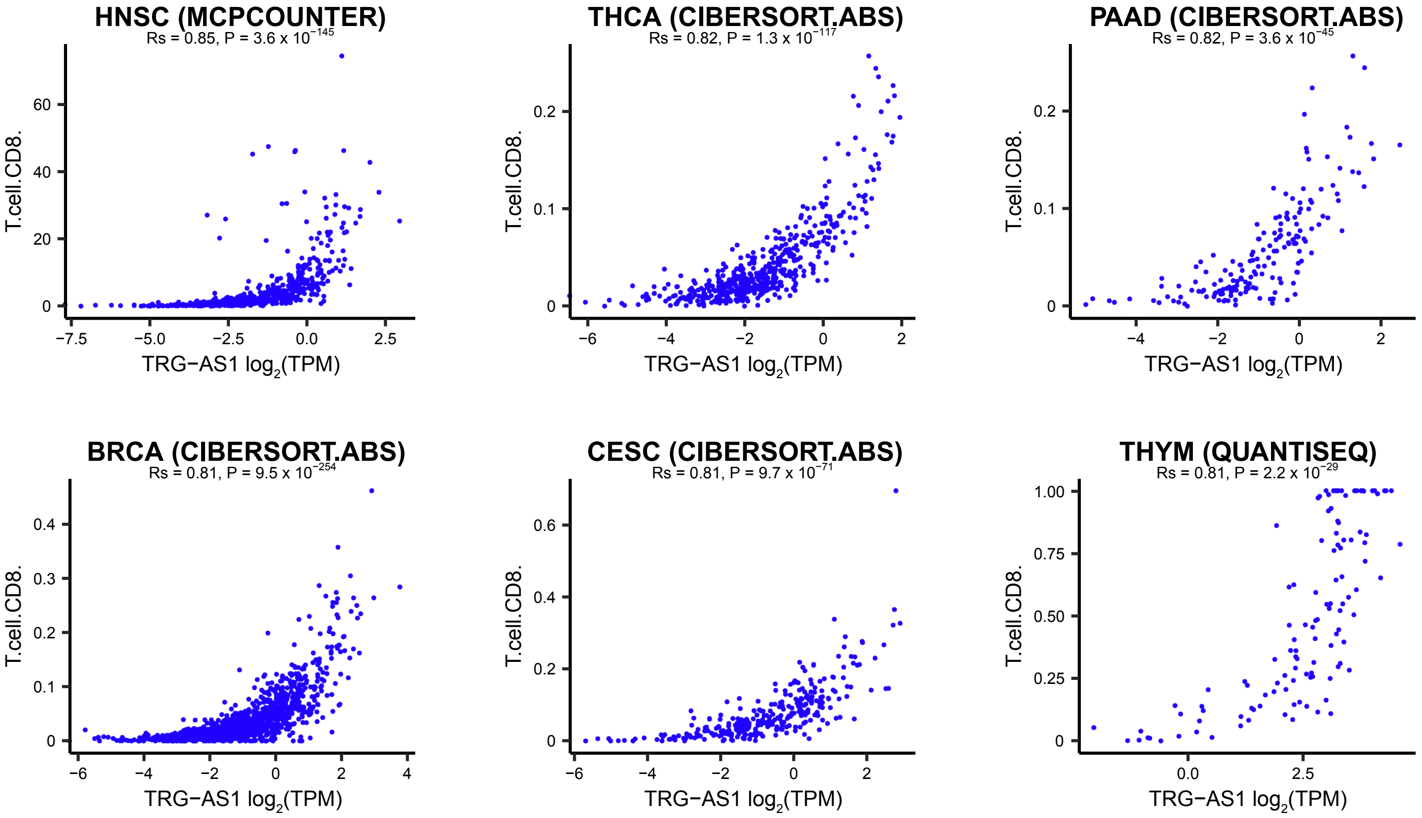

C. LncRNA LINC02446 positively correlated with CD8 T cells in multiple cancers

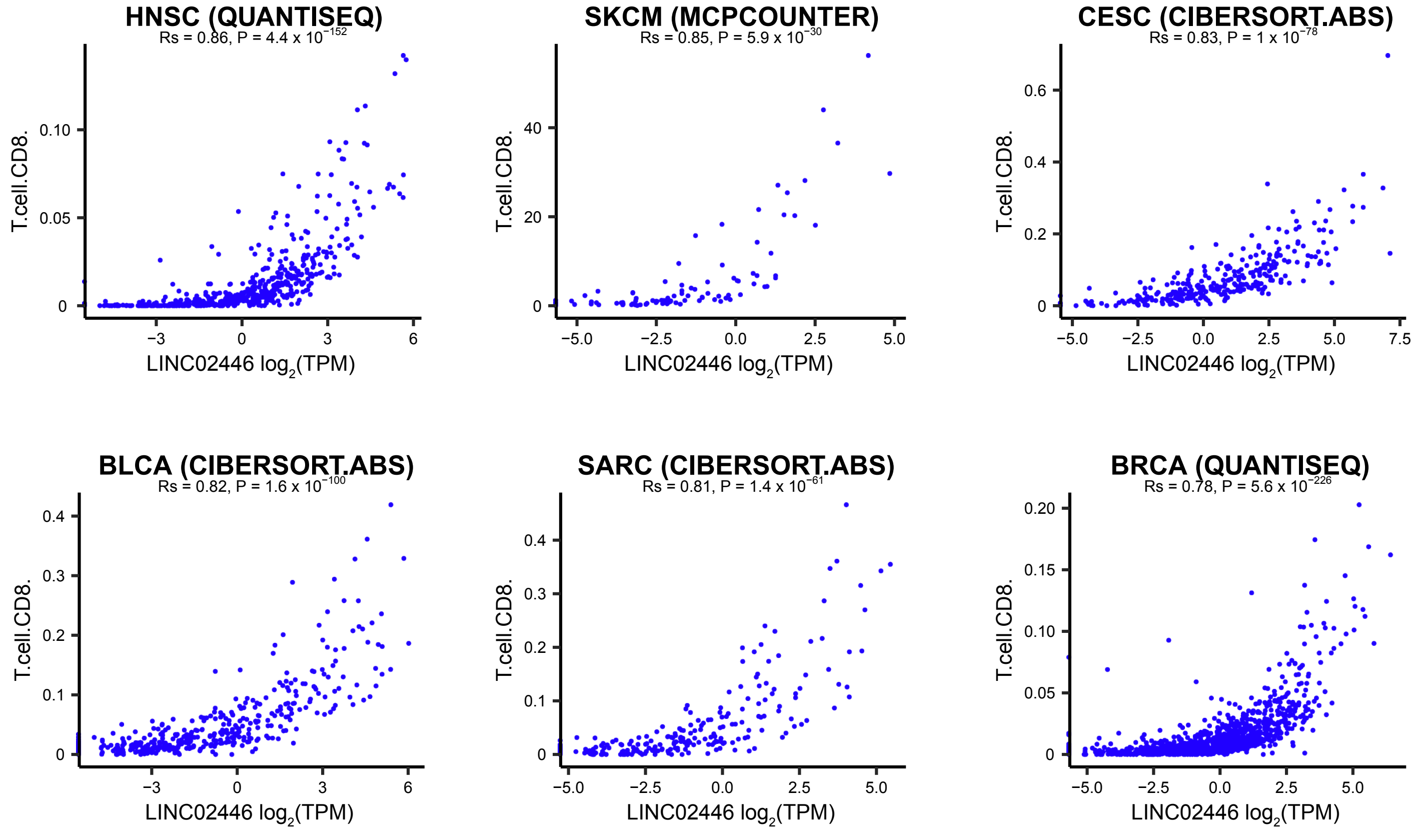

D. LncRNA HECW2-AS1 positively correlated with CD8 T cells in multiple cancers

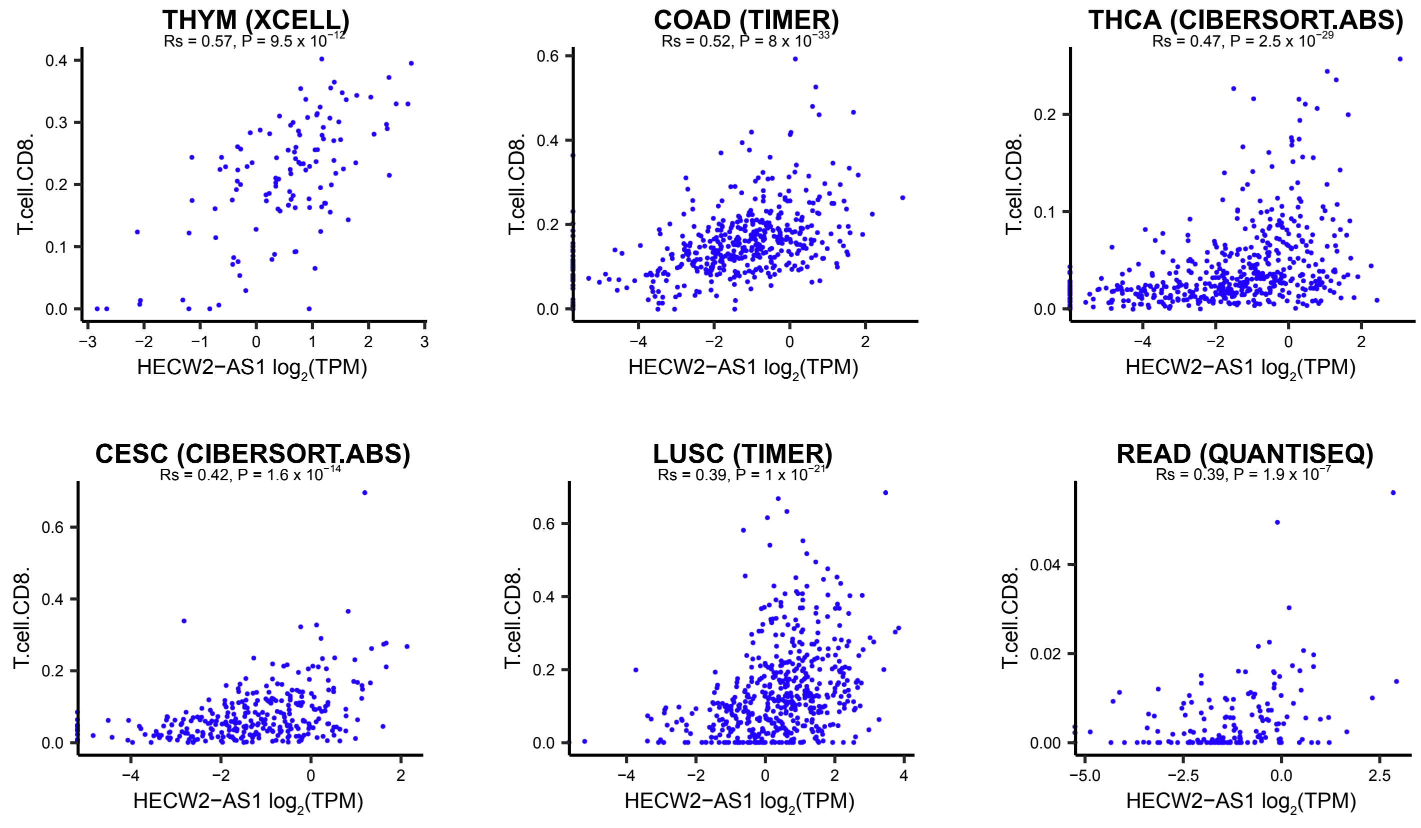

Supplementary Figure 6.

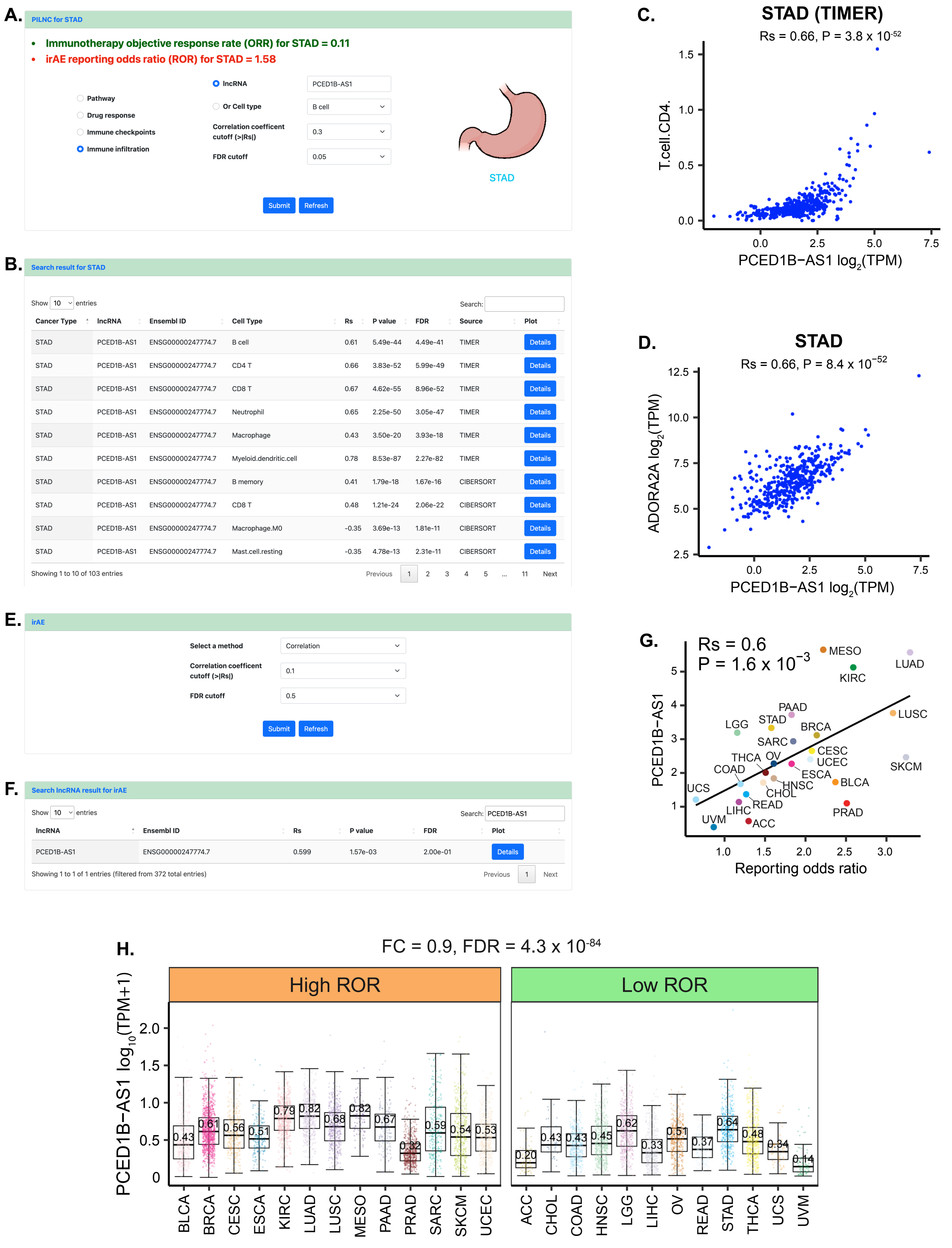

Supplement: Supplementary file 2 — Supporting Information [file ADVS-13-e13414-s001.pdf]
